# A comparison of three-factor structure models using WISC-III in Greek children with learning disabilities

**DOI:** 10.1186/s12991-018-0211-5

**Published:** 2018-10-03

**Authors:** Anna Adam, Grigoris Kiosseoglou, Grigoris Abatzoglou, Zaira Papaligoura

**Affiliations:** 1 School of Medicine, Service of Child and Adolescent Psychiatry, AHEPA Hospital, Third Psychiatric Clinic, Aristotle University of Thessaloniki, Thessaloniki, Greece; 2 School of Psychology, Aristotle University of Thessaloniki, Thessaloniki, Greece

**Keywords:** WISC-III, Factor structure, Learning disabilities, Comprehension, Picture arrangement

## Abstract

**Background:**

Children with learning disabilities are a heterogeneous group of children with a common characteristic discrepancy on the progress and development of their individual learning abilities. A few statistical analyses have been published regarding the factor analysis of the Greek Edition of Wechsler Intelligence Scale for Children-III. The aim of the research is the emergence of a new factorial model which describes the General Intelligence (g) of children and adolescents with learning disabilities, and that differs from the already existing intelligence models. This study aims to compare three-factor structure models of WISC-III in children with learning disabilities in the Greek population.

**Methods:**

A sample of 50 children were selected on the basis of research criteria from a total of 122 children who evaluated in a child psychiatric service in a general hospital, in a residential area in Greece. The Wechsler Intelligence Scale for Children—Third Edition was used to assess children’s cognitive function. Using multi-factor analysis, three alternative factor models were compared.

**Results:**

Analysis of factor structure models suggests a new bi-factorial model that more appropriately describes the areas of cognitive development of children with learning disabilities. The first factor includes Comprehension, Picture Arrangement, Coding, Block Design, and Object Assembly, whereas the second one combines Information, Similarities, Arithmetic, Vocabulary, and Picture Arrangement.

**Conclusions:**

The present study shows the existence of a factorial model with two factors: one aggregating the Comprehension verbal subtest with four performance subtests and the other the Picture Arrangement performance subtest with four verbal subtests. This two-factor model includes the loadings in two factors that relate to sequencing abilities and verbal reasoning abilities of children. These findings assert the clinical utility of the intelligence evaluation in the specific population.

## Background

The Wechsler Intelligence Scale for Children-Third Edition (WISC-III) is a widely used tool to measure intelligence that is systematically used for assessing children and adolescents with learning disabilities [1]. Children’s performance on the intelligence test guides the educational placement and determines the cognitive strengths and weaknesses of children.

The WISC-III factor structure of a group of children with special educational needs has been adequately studied and the data corroborate both the existence of a major factor for general intelligence and a two-factor model with verbal and performance factors [2]. The model proposed by Wechsler based on multiple-factor analysis is a model with four factors: (a) verbal comprehension, (b) freedom of distractibility, (c) perceptual organization, and (d) processing speed. According to researchers [3, 4], this model adequately describes the cognitive abilities of children with special educational needs. Wechsler also indicated that the four-factor model provides the best fit for multiple groups, such as a clinical sample of children with learning disabilities, reading disorders, or attention-deficit disorders [5].

The use of factor analysis models is a key tool in evaluating specific populations. Similar studies use special education samples, such as children with learning disabilities or other academic difficulties identifying four- and five-factor structure models on the WISC-III [4, 6–9]. Burton et al. [10] reported that the five-factor model concludes verbal comprehension, constructional praxis, visual reasoning, freedom from distractibility, and processing speed in a mixed clinical sample of 318 children. However, these factor models were contingent on the administration of the supplemental subtests.

Researches that examine WISC-III results in Greek special populations, such as children with mental retardation, organic disabilities, psychiatric disorders, or learning disabilities, are limited [11, 12]. The adaptation of the Greek scale is based on the British version of the WISC-III [5] and any modification made with the particularities of the Greek language and culture. Based on the analysis of the factor structure in WISC-III in the Greek weighting population, a three-factor model emerges, similar, to four-factor model proposed by Wechsler [13]. The Freedom from Distractibility Factor is not found, as well as in the Belgian–French Edition [14].

The present study aims to examine the factor structure of WISC-III and to compare three alternative factor models that best describe the intelligence of children and adolescents with learning disabilities in the Greek population. Because index scores are used for the interpretation of the test, it is critical to validate the factor structure of the WISC-III in the specific population. This study is an effort, for the first time in the Greek field, to detect the factor structure of the test in a clinical population. It is part of a wider research that scrutinizes the interaction of individual, family, social, and school factors in the performance of WISC-III in children with learning disabilities. The study of the scale’s factor structure in the particular population is an attempt to visualize the factor model that more precisely describes the areas of cognitive development.

## Methods

### Aim and study design

According to the above, we investigate: (a) the existence of a single-factor model which includes a major factor (g) for intelligence; (b) validation of the expected model on which the subtests information, similarities, arithmetic, vocabulary, and comprehension load on the verbal factor and the subscale picture completion, coding, picture arrangement, block design, and object assembly load on the performance factor; (c) the existence of a new modified factor model that fits better than the single or the expected factor model the data of the Greek sample studied.

The main objectives of the present study are expressed by the following research questions: Is there a strong structural factor that represents intelligence? Is the expected model verified? Multi-factor analyses are applied to children’s performances in WISC-III to point out the aggregation of the ten core subscales in individual meaningful factors.

### Participants

The total number of children and adolescents evaluated for their educational disabilities is 122. Out of the total of 122 children, 50 children were selected who met the following research criteria: (1) the initial referral was to evaluate the learning problem or the school failure of the child, (2) reported learning difficulties that met diagnostic criteria for learning disabilities, (3) a 3-year interval between two evaluations of the child with the WISC-III that meets the criteria for the high reliability of the scale in children with learning disabilities [15], and (4) the sample included children with severe psychopathology (pervasive developmental disorders, mental retardation, etc.) or organic diseases (neurological, endocrinological disorders, chronic diseases, etc.) who attend the service for evaluating their educational difficulties. The remaining 72 children did not meet the entry criteria, as there was insufficient evidence to perform a comprehensive psychometric assessment by administering the WISC-III.

The sample consisted of 50 children and adolescents (30 males and 20 females), who received comprehensive psychological evaluations for their learning disabilities. Their ages ranged from 7 to 15 years, and the average age of the participants was *M* = 11.5 years (SD = 2.05). From the total sample, 52% were elementary school students and 48% junior high and high school students. Of the participants, 74% attended schools in the urban area of Thessaloniki, 20% in suburban areas, and 6% attended schools in rural areas.

From the total sample, 58% of the children were diagnosed according to the criteria of the ICD-10 Classification of Mental and Behavioral Disorders [16] concerning Pervasive and Specific Developmental Disorders (F80–F89), and more specifically, the Phonological disorders, the Specific developmental disorders of scholastic skills, and the Pervasive developmental disorders. The rest of the children were diagnosed with Behavioral and Emotional Disorders (F90–F98), as well as the Intellectual Disabilities (F70–F79). From the total sample of children, 10% were students with high performance in school, 62% exhibited average performance, and 28% were children with school failure.

### Data collection

The study is part of a larger research that was carried out in the Child and Adolescent Service of the Third Psychiatric Clinic of the AHEPA University Hospital of Thessaloniki in Greece, from October 2010 to June 2016. The intelligence quotients of school aged children and adolescents were measured by the Greek version of the Wechsler Intelligence Scale for Children, Third Edition (WISC-III) [17]. The test was performed individually by an educational psychologist, and its duration was about 60–90 min. The scale consists of 13 subscales that assess the intellectual abilities; five core verbal, five core performance scales, two supplemental, and one optional subscale (*M *= 10; SD= 3). The first ten subscales deduce the Full Scale (FSIQ), verbal (VIQ), and performance (PIQ) intelligence indexes (*M *= 100; SD= 15). For research purposes, only the first ten mandatory subscales were administrated to participants.

### Statistical analyses

Scaled scores of the ten WISC-III subscales were analyzed using exploratory and confirmatory factor analysis. Exploratory factor analysis (EFA) was applied first to identify an initial factor structure for the underlying subtests. Then, confirmatory factor analysis (CFA) was used to validate specific patterns that have been revealed initially from EFA and to relate the observable scores of WISC-III subtests to factors. The goal of the multi-factorial exploratory and confirmatory analyses was to find the model that most efficiently describes the data structure.

EFA was applied using maximum-likelihood as extraction method that is considered the best one if data are relatively normally distributed [18]. We also used oblique rotation that allows the extracted factors to correlate. The sample size *n* = 50 of this research is considered marginally sufficient to yield a recognizable factor pattern [19, 20]. In addition, the subjects-to-variables ratio 50/10 = 5 meets the minimum requirement for EFA, because it is no lower than 5 [21]. Three well-known criteria were used to determine the number of factors to retain: Kaiser’s eigenvalue > 1 [22], Cattell’s scree test [23], and Horn’s parallel analysis [24]. All EFAs were performed using IBM SPSS Statistics 24 program.

Concerning CFA, the recent simulation studies showed that sample sizes of *n* = 50 participants are associated with satisfactory fit and reliable parameter estimates [25], especially in models with large factor loadings and high factor intercorrelations [26].

To evaluate the fit of the CFA models, we followed Hu and Bentler [27] guidelines for acceptable model fit: CFI and TLI values close to .95 or greater, SRMR values close to .08 or below, and RMSEA values close to .06 or below.

Finally, to compare the fit of non-nested CFA models, we calculated differences in BIC (Bayesian information criterion) values between models [28], while a Chi-square difference test was computed to compare nested models [29].

All CFAs were conducted using Mplus 5.0 program [30] based on maximum-likelihood estimation of parameters. It must be noted that Pearson correlation coefficient was used to calculate correlations among the WISC-III subtests.

## Results

Table 1 shows Pearson correlations among ten subtest scores as well as the values of skewness and kurtosis. As expected, all correlations are positive and significant (*p *< .01). We also observe that all values of skewness and kurtosis are between − 1 and +1, so we can conclude that the distribution of subtest scores fits the normal shape almost adequately.

**Table 1:** Measures of shape and Pearson correlations among WISC-III subtests

	*S*	*K*	IN	SI	AR	VO	CO	PC	CD	PA	BD
IN	.07	.36	–								
SI	.02	− .07	.69	–							
AR	− .40	− .01	.55	.69	–						
VO	− .10	− .09	.68	.75	.62	–					
CO	− .38	− .59	.54	.59	.65	.66	–				
PC	− .44	− .28	.39	.47	.62	.43	.56	–			
CD	− .39	− .56	.39	.39	.50	.51	.51	.51	–		
PA	− .58	− .45	.52	.58	.64	.59	.48	.45	.57	–	
BD	− .67	− .24	.45	.47	.65	.47	.56	.61	.49	.55	–
OA	− .67	− .19	.52	.49	.53	.49	.67	.60	.54	.51	.76

*S* skewness, *K* kurtosis, *IN* information, *SI* similarities, *AR* arithmetic, *VO* vocabulary, *CO* comprehension, *PC* picture completion, *CD* coding, *PA* picture arrangement, *BD* block design, *OA* object assembly

All correlations are significant (*p *< .01)

EFA was applied first to explore the factor structure for the underlying WISC-III core subtests. The Kaiser–Meyer–Olkin measure of sampling adequacy (KMO) was .89, indicating that EFA was appropriate for this sample. The results from Kaiser’s and Cattell’s criteria pointed to the presence of one dominant factor and another one, secondary and less important, while parallel analysis suggested a one-factor solution. We also considered the Chi-square goodness-of-fit test, produced from EFA. For the one-factor solution, it was found that *χ*^2^(35) = 56.33, *p *= .013, which means that there is additional significant amount of covariance among the subtest scores after one factor has been extracted. In contrary, for the two-factor solution, the test was not significant (*χ*^2^(26) = 24.34, *p *= .556), indicating that the sample data are likely to have arisen from two correlated factors. Thus, we decided to adopt, as initial factor structure of the ten subtests, the two-factor solution. The pattern matrix presented in Table 2 displays the rotated factor loadings of the ten subtests for one- and two-factor solution. After inspecting the pattern of loadings for the two-factor solution, and setting the cutoff at .41, it was found that the first factor was mainly defined by the performance subtests except “Picture Arrangement” which had higher loading on the second factor, while the second factor was loaded mainly by the verbal subtests except “Comprehension” which had higher loading on the first factor.

**Table 2:** Exploratory factor analysis

	One factor	Two rotated factors	
		Factor 1	Factor 2
Information	*.71*	.03	− *.75*
Similarities	*.78*	− .06	− *.92*
Arithmetic	*.82*	.38	− *.50*
Vocabulary	*.79*	− .03	− *.89*
Comprehension	*.79*	*.44*	− .41
Picture completion	*.68*	*.69*	− .05
Coding	*.64*	*.50*	− .19
Picture arrangement	*.73*	.33	− *.45*
Block design	*.74*	*.93*	.10
Object assembly	*.75*	*.87*	.03
Eigenvalue	5.99	5.99	1.01
Variance (%)	59.89	59.89	10.09

Maximum-likelihood–direct oblimin factor loadings for one- and two-factor solution

Loadings > |.41| highlighted in italic

Next, to validate the pattern of two-factor loadings proposed by EFA, CFA was conducted in the following way: first, a CFA was used to test the fit of the model with two correlated factors (M2) resulting from EFA. Then, a one-factor model (M1) in which all subtests loaded on a single factor was considered, and a Chi-square difference test for nested models was computed to compare M1 and M2 models to decide whether the one fitted significantly better or worse than the other. Finally, a third CFA was conducted to test the fit of the expected two-factor model (M3) in which all five performance subtests load on a factor (performance factor), while all five verbal subtests load on another one (verbal). The two non-nested models M3 and M2 were compared via Bayesian information criterion, BIC index. The model with the smallest BIC value is considered to be the best model.

After inspection of modification indices given by Mplus program, we allowed a correlation between the errors terms of Block Design and Object Assembly subtests for the three models. Table 3 contains fit indices for the three alternative models. We observe that model M2, corresponding to the two correlated factor solution proposed by EFA fits the data very well: *χ*^2^ = 38.39, *df* = 33, *p *= .24, CFI = .98, TLI = .98, RMSEA = .057, and SRMR = .050. The correlation between factors was *r *= .85, *p *< .001. Standardized loadings (Table 4) range from .66 to .85 supporting an internally consistent solution, and are all significant (*p *< .001).

**Table 3:** Summary of fit indices for three alternative CFA models

Models	*χ* ^2^	*df*	*p*	CFI	TLI	RMSEA	SRMR	BIC
Single-factor (M1)	49.94	34	.038	.95	.93	.097	.059	2465.1
Correlated two-factor (M2)^a^	38.39	33	.238	.98	.98	.057	.050	2457.5
Correlated two-factor (M3)^b^	41.58	33	.145	.97	.96	.072	.053	2460.7

^a^M2: factor 1 is loaded by performance subtests except “Picture Arrangement” which loads on the second factor; factor 2 is loaded by verbal subtests except “Comprehension” which loads on the first one

^b^M3: factor 1 is loaded by performance subtests; factor 2 is loaded by verbal subtests

**Table 4:** Confirmatory factor analysis

Subtests	Factor 1	Factor 2	R^2^
Comprehension	.81		.66
Picture completion	.73		.53
Coding	.66		.44
Block design	.75		.56
Object assembly	.79		.63
Information		.76	.58
Similarities		.85	.73
Arithmetic		.81	.65
Vocabulary		.84	.70
Picture arrangement		.72	.52

Correlated two-factor model; standardized loadings

All loadings are significant (*p *< .001)

For the one-factor model M1, we observe (Table 3) that it fits the data well but worse than model Μ2: *χ*^2^ = 49.94, *df* = 34, *p *= .038, CFI = .95, TLI = .93, RMSEA = .097, and SRMR = .059. Furthermore, according to the Chi-square difference in the fit between the nested models M1 and M2: Δχ^2^ = χ^2^(M1) − χ^2^(M2) = 11.55, Δ*df* = *df*(M1) − *df*(M2) = 1, *p *< .001, it was found that model M1 fits the data significantly worse than model M2.

Finally, model M3 fits the data satisfactory (Table 3) but slightly worse than model M2: *χ*^2^ = 41.58, *df* = 33, *p *= .15, CFI = .97, TLI = .96, RMSEA = .072, and SRMR = .053. To compare the non-nested models M2 and M3, we calculated the difference in BIC values between the two models: BIC(M3)–BIC(M2) = 2460.7–2457.5 = 3.2. According to Kass and Raftery [28], difference in BIC of 2–6 points is considered as positive evidence in favor of the model with the smaller BIC. Hence, we can conclude that model M2 fits the observed data better than model M3.

## Discussion

Findings from this study demonstrated that the (g) factor reflecting General Intelligence, as emerged from a single-factor model, remains a major factor for children with learning disabilities. These findings are consistent with the previous surveys. According to Poulson and Scardapane, the scale’s factor structure in a population of individuals with special educational needs has confirmed the existence of a powerful factor for General Intelligence and partial two-factor models, the Verbal and Performance Intelligence Scale [2].

However, adopting a single-factor model does not allow us to better understand the cognitive abilities of the children while taking into account individual variations in their performance on each of the two scales. Even though the single-factor model provides information on children’s cognitive potential, it does not allow researchers to deepen their intrapersonal profile. These findings are in agreement with Konold et al. [3] and Grice et al. [4] which conclude in models with more than one factor.

The expected model has been validated as a sufficiently good illustrative model, as anticipated, however, a new alternative model emerges that outweighs it and better fulfills the statistical criteria. The new proposed two-factor model better adapts to General Intelligence of children with learning disabilities. This model consists of two new factors. The first factor that relates to the sequencing abilities of children consists of four performance subtests and one verbal subtest: picture completion, coding, block design, object assembly, and comprehension. The second factor, which relates to the verbal reasoning abilities of children, consists of four verbal and one performance subtests: information, similarities, arithmetic, vocabulary, and picture arrangement. This new two-factor model fits more adequately than both the single-factor model and the expected model.

This finding could be particularly noteworthy, as it is different from any similar research, probably due to the correlation observed between comprehension (verbal subtest) and picture arrangement (practical subtest). Krippner [31], Brannigan [32], and Beebe et al. [33] point out that these two subtests are indicators of measuring social competence and social maturity. In addition, Rapaport et al. [34] emphasize that Comprehension and Picture Arrangement can be considered complementary, voicing common abilities in relation to social understanding.

At the same time, Sattler [35] reported that Comprehension measures the child’s social knowledge and the level of social maturation and Picture Arrangement evaluates the child’s ability to comprehend and evaluate social situations. The correlation of these two connotations led to their association as a measurement of “social intelligence” [36]. The relevance of the two subtests may partially explain this new model. In addition, there is no absolute agreement between researchers about linking these two abstracts to the measurement of “social intelligence” [37]. This discrepancy is related to the variety in the definition of social competence and maturity [33].

One possible explanation might be that even though Picture Arrangement is a performance subtest, it can also measure the ability for verbal sequencing in children that have well-developed verbal skills [5]. This finding is asseverated by the clinical practice, as well as the children who understand and tell the story correctly in picture arrangement subtest to achieve better results than the children who fail in understanding the content of the stories. This hypothesis, however, needs further investigation.

Giannitsas and Mylonas [13] conducted factor analyses in the weighting Greek sample and resulted in three factors, verbal comprehension, perceptual organization, and processing speed. These results are not validated in the present study; however, the majority of surveys measure children’s performance in 13 WISC-III subtests (primary and complementary), while our study is based on the ten core endorsements. A key advantage is that it is the first study that examines the factor structure of WISC-III in a clinical population in children of Greece.

However, there are certain limitations, concerning the heterogeneous nature of the sample, which consisted of children with a variety of diagnoses. In addition, a larger sample would allow for separate factor analyses across different clinical populations to determine if the findings of this study are replicable. Although the sample of the survey fulfills the research’s criteria, a larger sample would allow the conclusions to be strengthened. For example, individual diagnostic categories in the group of children with learning disabilities could be evaluated. In addition, the factor structure of the scale could be examined in different age and sex groups.

## Conclusions

The new proposed model follows the widely used model of two individual factors (VIQ and PIQ); however, it has some significant differences, as Performance and Verbal subscales cannot be allocated equally to the two factors, but they cross each other. This study provides evidence that the first factor relates to the sequence abilities of children, the ability to think with logical sequences, rationally, and to perceive the elements in a specific order. These skills are very important in comprehension, and written and oral language. The second factor related to verbal reasoning abilities focuses on the ability of children to think in a structured way and to comprehend concepts composed by words. These skills are an essential element of the learning process.

These results may provide useful information to psycho-educational assessments and improve educational planning and therapeutic interventions in children with learning disabilities in the Greek population. Nevertheless, it is critical to further investigate the meaning and interpretation of these factors in clinical populations.
